# LncRNA HOXA-AS2 promotes glioblastoma carcinogenesis by targeting miR-885-5p/RBBP4 axis

**DOI:** 10.1186/s12935-020-01690-1

**Published:** 2021-01-11

**Authors:** Jixin Shou, Haidong Gao, Sen Cheng, Bingbing Wang, Haibo Guan

**Affiliations:** Department of Neurosurgery, The Fifth Affiliated Hospital of Zhengzhou University, Erqi District, No. 3 Kangfu Front Street, Zhengzhou, 450052 Henan China

**Keywords:** Glioblastoma, HOXA-AS2, miR-885-5p, RBBP4, Carcinogenesis

## Abstract

**Background:**

LncRNA HOXA-AS2 has been found in the literature to deteriorate glioblastoma. However, its regulatory mechanism is yet to be fully investigated. Our study focused chiefly on the interaction and role of the HOXA-AS2/miR-885-5p/RBBP4 axis in the development of glioblastoma.

**Methods:**

qRT-PCR analysis was performed to detect the expression of lncRNA, miRNA and mRNA in glioblastoma tissues and cells. Dual-luciferase assay, RIP assay and RNA pull-down assay were later carried out to reveal the interactions among HOXA-AS2, miR-885-5p and RBBP4. After that, CCK-8 assay, BrdU assay, nude mice xenografting assay, western blot assay, and flow cytometry were carried out to analyze the effect of the HOXA-AS2/miR-885-5p/RBBP4 axis on glioblastoma samples.

**Results:**

HOXA-AS2 and RBBP4 were found to be overexpressed in glioblastoma. Experimental results showed that HOXA-AS2 and RBBP4 contributed to the tumorigenesis of glioblastoma cells. However, miR-885-5p was observed to be downregulated in glioblastoma. Findings also indicated that HOXA-AS2 could negatively regulate miR-885-5p, thereby enhancing RBBP4 expression.

**Conclusion:**

Overall, HOXA-AS2 promoted the tumorigenesis of glioblastoma by targeting and regulating miR-885-5p to induce the expression of RBBP4.

## Background

Glioblastoma can be described as an aggressive tumor whose origin can be traced to the brain or spinal cord [1]. Because of the complexity of the nervous system and the limitations of surgical operations, hundreds of thousands of patients with glioblastoma have lost their lives [2]. It is even more difficult to treat this cancer as the blood–brain barrier prevents chemicals from entering the brain, thereby resulting in low survival rates, high relapse rates, and poor prognosis rates for glioblastoma patients [3]. Although radiotherapy, chemotherapy and other surgical techniques have been used to treat glioblastoma patients [4–7], they do not guarantee the long-term survival of victims. By investigating the underlying pathogenesis of glioblastoma, it will be feasible to discover new diagnoses and treatment methods that can improve the chances of survival of glioblastoma patients in the long-term.

Long non-coding RNAs (lncRNAs) can be referred to as non-coding RNA molecules with more than 200 nucleotides. HOXA cluster antisense RNA 2 (HOXA-AS2), a long non-coding RNA, is directly involved in such cellular processes as cell proliferation and gene expression. Over the years, HOXA-AS2 has been linked to human carcinogenesis [8]. HOXA-AS2 has even been found to play a carcinogenic role in the pathophysiology of various cancers, including pancreatic cancer, non-small cell lung cancer, and osteosarcoma [9–11]. One study reported that HOXA-AS2 was upregulated in cells with colorectal cancer [12]. Apart from the fact that HOXA-AS2 expression was proved to be upregulated in glioma, silencing HOXA-AS2 promoted the growth of glioma [13]. This study aimed to demystify the regulatory mechanism of HOXA-AS2 in glioblastoma.

Regarded as small non-coding RNAs encoded by endogenous genes, miRNAs participate actively in transcriptional repression or RNA degradation by binding to target genes to repress cell expression. Studies have shown the involvement of miRNA in the biological processes of many forms of cancers [14]. Moreover, the differential expression of miR-885 provides novel therapeutic strategies and diagnostic biomarkers for cancers [15, 16]. Most of the research articles in the literature have confirmed that miR-885-5p plays diverse roles in different cancers. For instance, miR-885-5p acted as a tumor promoter and tumor suppressor in hepatocellular carcinoma and colorectal cancer, respectively [17, 18]. While the overexpression of miR-885-5p impeded cell invasion in glioma cells [19, 20], researchers are yet to investigate the upstream regulator of miR-885-5p in glioblastoma.

LncRNAs have the potential to sequester and release miRNAs from specific mRNA targets, thereby modulating the expression and biological functions of miRNAs [21, 22]. In glioma, miR-885-5p is often used as the upstream miRNA of target genes [19]. RBBP4, which is located on chromosome 1p35.1, consists of 13 exons. It also encodes RBBP4, which belongs to a highly conserved subfamily of WD-repeat proteins. After predicting the target genes of miR-885-5p using miRDB and analyzing the potential protein–protein interaction with STRING, we found that RBBP4 displayed credible results. The role of RBBP4 in cancer has been explored in the literature, and RBBP4 was found to be a pro-oncogenic factor during gastric carcinogenesis [23]. RBBP4 upregulation also appeared in the hepatic metastasis of colon cancer, which provided a molecular basis for the diagnosis of the disease [24]. In addition to that, silencing RBBP4 attenuated GC cell growth and increased cell apoptosis [23]. However, it is unclear whether RBBP4 plays a significant role in glioblastoma and whether it can be regulated by miRNAs. Hence, our study aimed to unravel the role of the HOXA-AS2/miR-885-5p/RBBP4 axis in glioblastoma. We believed that the outcome of this research might provide insights into understanding the pathological processes involved in the occurrence of glioblastoma.

## Materials and methods

### Bioinformatics analysis

Online tool miRDB was used to predict the target genes of miR-885-5p. The upregulated DEGs were screened out using GEPIA, with log2|FC|> 2 and adjusted P < 0.01. Venny 2.1.0 was then leveraged to overlap the common genes from miRDB and GEPIA. Finally, the common genes screened were subsequently uploaded to STRING (https://string-db.org) for protein–protein interactions analysis.

### Sample acquisition and cell culture

Glioblastoma tissues and corresponding adjacent normal tissues were collected from 33 patients at the Fifth Affiliated Hospital of Zhengzhou University. The characteristics of 33 patients with glioblastoma are listed in Table 1. Our study was approved by the Ethics Committee of the Fifth Affiliated Hospital of Zhengzhou University. All cell lines were obtained from the BNCC (Beijing, China), such as glioblastoma cell lines (U251, U87, A172, SHG44 and SNB19) and the normal human astrocytes cell line (NHA). U251, U87, A172 and SNB19 cells were cultured in DMEM-H (Cat#: E600004, Sangon, China) with 10% fetal bovine serum and 100 U/mL streptomycin under 5% CO_2_ at 37 °C. The SHG44 cell line was cultured in RPMI-1640 (Cat#: E600028, Sangon, China) with the same culture conditions as other cell lines.

**Table 1:** Baseline characteristics of 33 patients with glioblastoma

Total no. patients = 33	No. (%)
Age at diagnosis (years)	
Age > 55	18 (54.55)
Age ≤ 55	15 (45.45)
Gender	
Female	20 (60.61)
Male	13 (39.39)
Tumor origin	
Primary	23 (69.7)
Secondary	10 (30.3)
Tumor localization	
Left hemisphere	11 (33.33)
Right hemisphere	14 (42.43)
Both hemisphere	8 (24.24)
Tumor volume (median, range)	17.9 cm^3^ (1.2–57.8)

### Cell transfection

The small interfering RNAs (siRNAs) of HOXA-AS2 (si-HOXA-AS2) and RBBP4 (si-RBBP4), miR-885-5p mimic, miR-885-5p inhibitor and negative control (NC) were synthesized and provided by GenePharma (Shanghai, China). Using Lipofectamine 2000 (Cat#: 11668019, Thermo Fisher Scientific, USA) at room temperature for 4 h, the cells were transfected with 50 nM si-HOXA-AS2, si-RBBP4, miR-885-5p mimic and miR-885-5p inhibitor. After incubation for 2 days at 37 °C, the transfected cells were collected to detect the transfection efficiency using qRT-PCR.

### qRT-PCR

The total RNAs from 33 clinical tumor tissue samples, corresponding non-tumor tissue samples or cells, were separated using TRIzol Reagents (Cat#: 15596026, Thermo Fisher Scientific, USA). The isolated RNAs were subsequently reverse-transcribed into cDNA after detecting RNA concentration. The miRVana qRT-PCR miRNA Detection Kit (Cat#: AM1558, Thermo Fisher Scientific, USA) was utilized to reverse-transcribe miRNAs into cDNA according to the user’s manual. The SuperScript III First-Strand Synthesis SuperMix for qRT-PCR (Cat#: 11752050, Thermo Fisher Scientific, USA) was used to reverse-transcribe the lncRNAs and mRNAs into cDNA. After synthesizing cDNA, qRT-PCR was performed using the StepOnePlus Real-Time PCR System (Cat#: 4376600, Thermo Fisher Scientific, USA). The data of qRT-PCR were analyzed with the 2^−ΔΔCt^ method. GAPDH was used as the internal control of mRNAs and lncRNAs, while U6 was utilized as the reference control of miRNAs. The sequences of the primers are illustrated in Table 2.

**Table 2:** Primer sequences for RT-qPCR

Gene name	Primer-F (5′–3′)	Primer-R (5′–3′)
HOXA-AS2	AACCCATCTTTGCCTTCTGC	CGGAGGAGTTTGGAGTTGG
miR-885-5p	GTCCATTACACTACCCTGCCTC	CGCGAGCACAGAATTAATACG
U6	CTCGCTTCGGCAGCACA	AACGCTTCACGAATTTGCGT
RBBP4	GTAGAGAGCTCTTCAGCAAGAC	AGGAACAGCTGGAGGAAATG
GAPDH	GGGAGCCAAAAGGGTCAT	GAGTCCTTCCACGATACCAA

### Subcellular fractionation location

The PARIS Kit (Cat#: AM1921, Thermo Fisher Scientific, USA) was used to separate nuclear and cytoplasmic RNAs. The isolated RNA products were analyzed using qRT-PCR. The GAPDH served as the cytoplasmic control, while U2 was used as nuclear control.

### CCK-8 assay

The effect of the HOXA-AS2/miR-885-5p/RBBP4 axis on cell viability was assessed using the CCK-8 assay. Transfected U87 and U251 cells (100 μL) in the logarithmic growth phase were seeded into the 96-well plates at a density of 2000 cells/well before being incubated at 37 °C. After an incubation period of 0, 24, 48 and 72 h, 10 μL CCK-8 solution was added to each well. The cells were later incubated for 2 h according to the instruction manual of the CCK-8 Kit (Cat#: E606335, Sangon, China). The absorbance was measured at 450 nm with a microplate reader to assess cell viability.

### BrdU assay

The BrdU Cell Proliferation ELISA Kit (colorimetric) (Cat#: ab126556) was purchased from Abcam (UK) for BrdU assay analysis. The transfected cells (1 × 10^5^) were added to each 96-well plate. Then, 20 μL BrdU was added to each well and incubated for 24 h. After incubation, the cells were fixed using a 200 μL/well fixing solution for 30 min. The cells removed from the fixing solution were later incubated with 100 μL/well anti-BrdU antibody for 1 h at room temperature. Next, 100 μL/well Peroxidase Goat anti-mouse IgG was added to the cells and then incubated for 30 min at room temperature. Finally, the absorbance was measured at 450 nm to assess the ability of the cells to proliferate.

### Cell adhesion assay

Type I collagen (10 μg/mL) or 10% bovine serum albumin was used to pre-coat 96-well plates overnight. Next, 5 × 10^3^ cells/well transfected cells were added to the pre-coated 96-well plates and then incubated for 1 h. After the incubation period, the non-adherent cells were removed, and the adherent cells were incubated with a recovery medium. Finally, 10 μL MTT was added to cells and incubated for another 2 h. The absorbance was measured at 570 nm to evaluate cell adhesion.

### Cell apoptosis detection

The cells transfected were resuspended in pre-chilled PBS and then diluted to 1 × 10^6^ cells/ml using 1 × Annexin binding buffer. Then, 5 µL of the Annexin V-FITC staining solution and 5 µL of 100 µg/ml PI staining solution was added to the cells and then incubated in the absence of light for 30 min. Finally, the apoptosis rate was detected using flow cytometry. This was done according to the manufacturer's instructions.

### Nude mice xenografting assay

Female BALB/c nude mice 6–8 weeks of age (Beijing Vital River Laboratory Animal Technology Co., Ltd.) were intracranial injected with 3 × 10^4^ U87 cells transfected with HOXA-AS2 knockdown recombinant lentivirus (Si-LNC) or the negative control (NC). The injection was carried out according to the methodology used in previous studies [25]. The growth of tumors in vivo was monitored by biophotonic imaging using a Xenogen IVIS 200 system (Xenogen, Palo Alto, CA). Images were captured and quantified with Xenogen Living Image 4.1 software. The image intensities were expressed as photon flux per second per square centimeter and steradian. After the nude mice were killed on day 29, the tumors were resected and subjected to HE pathology analysis.

### Dual-luciferase assay

The segments of wild-type HOXA-AS2 and RBBP4 3′UTR containing the binding site of miR-885-5p and the segments of mutant HOXA-AS2 and RBBP4 3′UTR without the binding site of miR-885-5p were inserted into pmirGLO vectors. The corresponding recombinant vectors included pmirGLO-WT-HOXA-AS2, pmirGLO-MUT-HOXA-AS2, pmirGLO-WT-RBBP4, and pmirGLO-WT-RBBP4. These recombinant vectors were then co-transfected with miR-885-5p mimic or NC into U87 and U251 cells. After a transfection period of 48 h, the luciferase activity was assessed with a dual-luciferase reporter assay system (Promega, USA).

### RNA immunoprecipitation (RIP) assay

The interaction between HOXA-AS2 and miR-885-5p was further identified using RIP assay. The U87 and U251 cells transfected with miR-885-5p mimic were then cultured for 48 h. Next, the transfected cells were collected using trypsin and then lysed with RNA lysis buffer. After the cells were lysed, the cell lysates were incubated with magnetic beads conjugated to anti-Argonaute2 (AGO2) or IgG1 as a negative control. After washing the unbound material on the magnetic beads with RIP buffer and PBS, the magnetic beads were resuspended with TRIzol Reagents (1 ml) to purify the bound RNAs. The extracted RNAs were eventually analyzed by qRT-PCR.

### RNA pull-down assay

The interaction between RBBP4 and miR-885-5p was identified using an RNA pull-down assay. The cells (6 × 10^5^/well) were seeded in 6-well plates and incubated overnight. Then, the biotinylated negative control (Bio-NC) and biotinylated miR-885-5p mimic (Bio-miR-885-5p) bought from RiboBio (China) were transfected with the cells using lipofectamine 2000. After 48 h, the cells were incubated with streptavidin beads for 3 h. Finally, qRT-PCR analysis was performed to measure RBBP4 expression.

### Western blot assay

The total proteins were extracted using a RIPA lysis system (Cat#: C500005, Sangon, China). Next, the proteins were separated with 10% SDS-PAGE gel and then transferred to PVDF membranes (Cat#: F019532, Sangon, China). After blocking the membranes in 5% skim milk, the hybridization membrane was incubated at 4 °C overnight with primary antibodies RBBP4 (Cat#: D154089, Sangon, China), Twist (Cat#: ab49254, Abcam, UK), Slug (Cat#: ab51772, Abcam, UK), Vimentin (Cat#: ab92547, Abcam, UK), MMP-2 (Cat#: ab92536, Abcam, UK) and GADPH (Cat#: D190090, Sangon, China). The membranes were then incubated with the corresponding secondary antibody for 2 h. Subsequently, the protein was detected using the ECL chemiluminescence kit (Cat#: C510043, Sangon, China) according to the manufacturer’s instructions.

### Statistical analysis

The data were represented in the form of mean ± standard deviation (SD), and three independent data were collected for each experiment. The Student's *t*-test was used to statistically analyze two groups, while the one-way analysis of variance (ANOVA) test was used to statistically evaluate multiple groups. P < 0.05 was considered to be statistically significant.

## Results

### The identification of HOXA-AS2/miR-885-5p/RBBP4 interactome as the study object

**Fig. 1:**
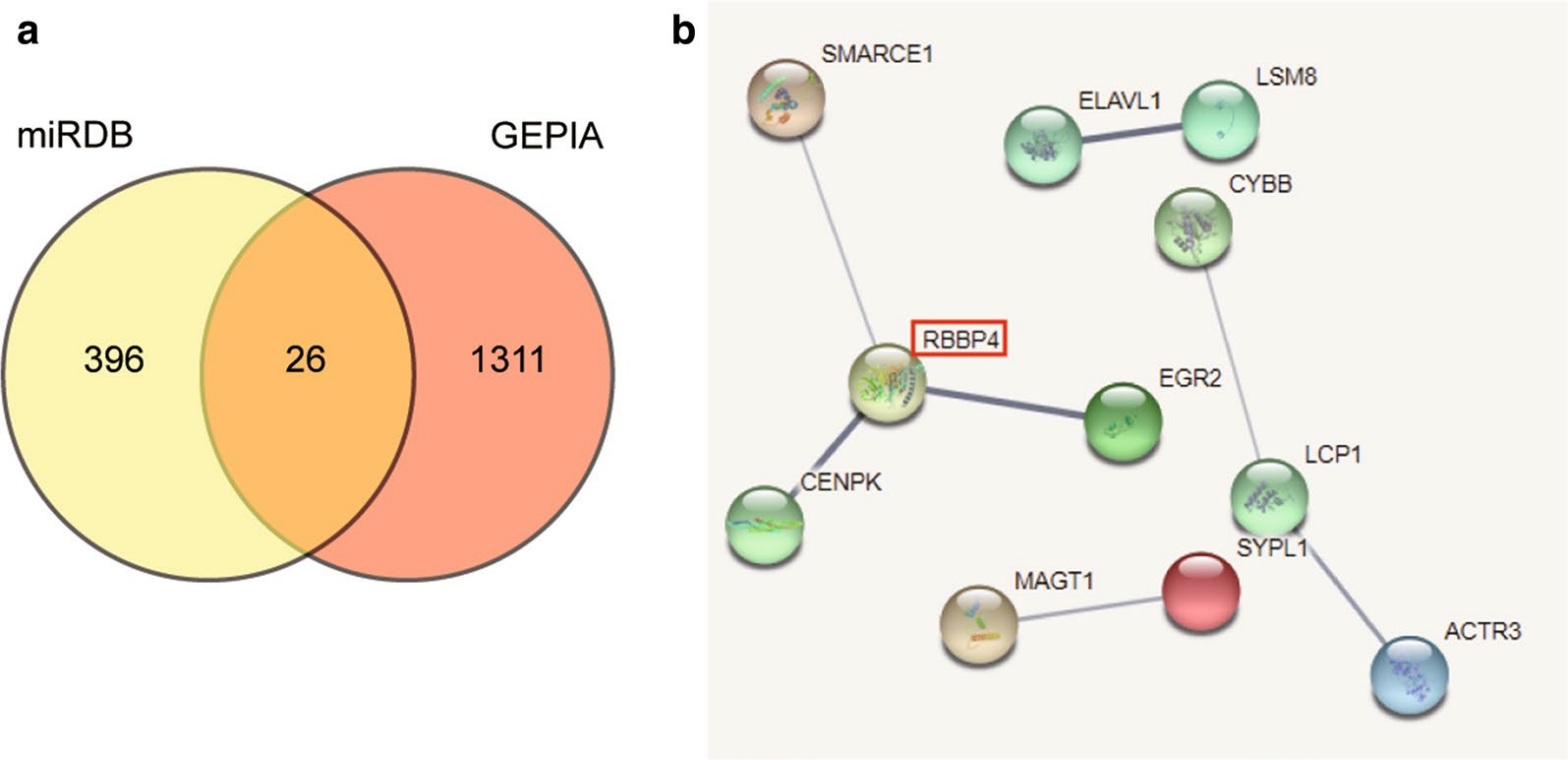
RBBP4 was identified as our gene of interest to be investigated. **a** Twenty-six genes were overlapped from miRDB and GEPIA using Venny 2.1.0. Online tool miRDB was used to predict the target genes of miR-885-5p. GEPIA was utilized to select the desired DEGs with log_2_|FC|> 2 and adjusted P < 0.01. **b** RBBP4 was the key gene connecting three other genes by STRING analysis. STRING was leveraged to analyze the potential protein–protein interaction of 26 genes

HOXA-AS2 has been confirmed to promote the metastasis of glioma [13]. We intended to expand the interacting network that involves HOXA-AS2 in glioblastoma. By interrogating the predicted 24 downstream miRNAs from ENCORI starbase database, we found that miR-885-5p, the most highly scored miRNA, was once confirmed to be a suppressor in glioma, using U87 and U251 cell line as study models [19, 20]. To identify the downstream effectors of miR-885-5p, we intersected the predicted target genes from the miRDB database and the differentially expressed genes (DEGs) from GEPIA using the criteria of log_2_|FC|> 2 and adjusted P < 0.01. The intersected 26 genes were uploaded to STRING to analyze the potential protein–protein interactions among them (Fig. 1a). RBBP4 showed a very high confidence level, and it connected with three other genes (Fig. 1b). Besides, RBBP4 disruption was found to suppress glioblastoma growth in vivo [26] and was confirmed to be a tumor promoter in gastric cancer samples [23]. The underlying mechanism of this suppression remained unknown, and the upstream regulators of RBBP4 remained a mystery. We herein identified a novel interactome in glioblastoma even though the effects of this interactome in glioblastoma has never been studied.

### Effects of Si-HOXA-AS2 on glioblastoma samples

**Fig. 2:**
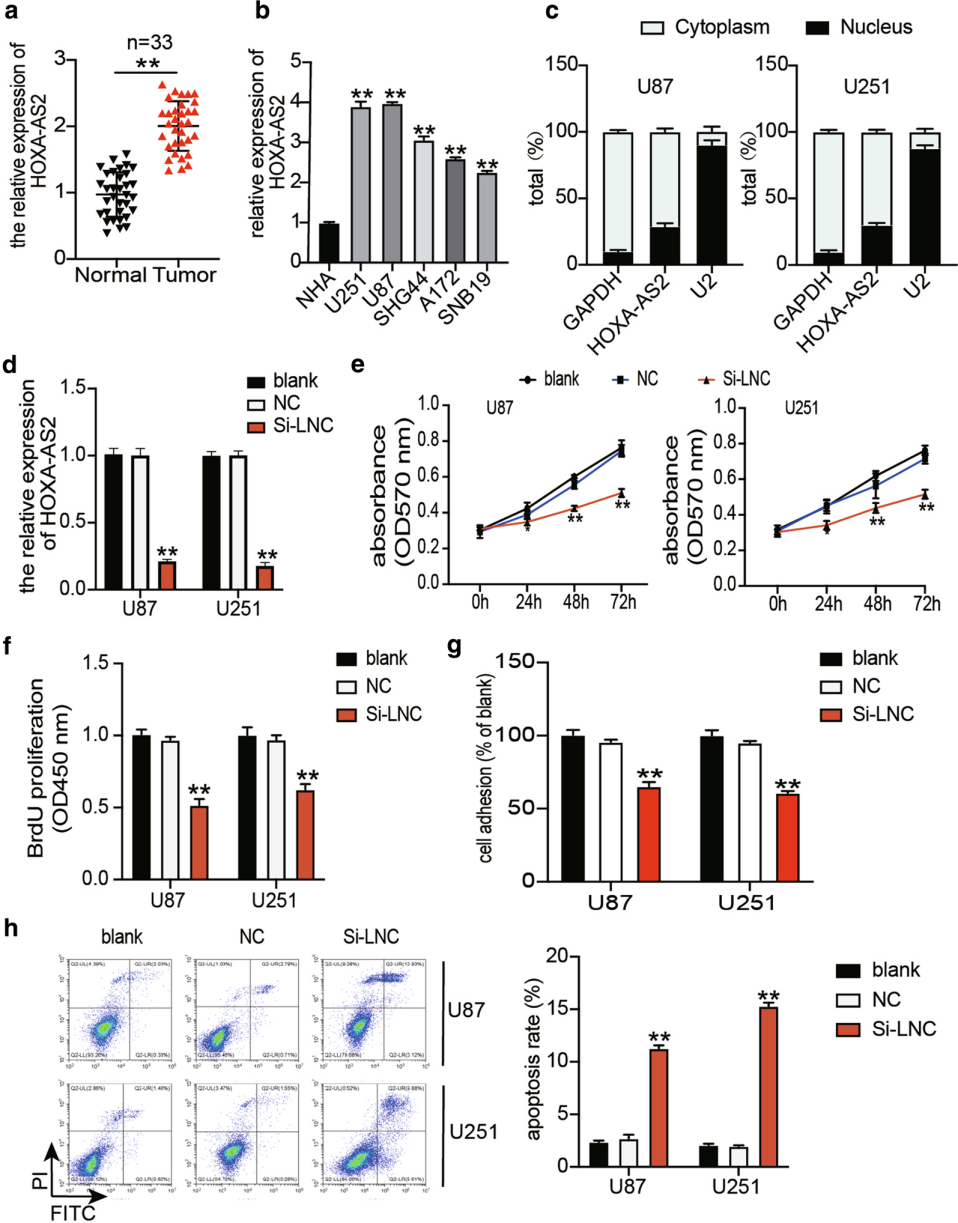
Si-HOXA-AS2 inhibited cell viability, cell proliferation and cell adhesion, but it induced cell apoptosis in glioblastoma cells. **a** HOXA-AS2 expression in glioblastoma tissues and non-tumor tissues was analyzed using qRT-PCR. N = 33. **b** HOXA-AS2 expression in glioblastoma cell lines (U251, U87, A172, SHG44 and SNB19) and normal human astrocytes cell line (NHA) was analyzed using qRT-PCR. **c** The intracellular distribution of HOXA-AS2 was identified using a subcellular fractionation location assay. **d** The transfection efficiency of si-HOXA-AS2 was verified in U87 and U251 cell lines by qRT-PCR. **e** The viability of the transfected U87 and U251 cells was measured after performing the CCK-8 assay. **f** The proliferation of the transfected U87 and U251 cells was measured using the BrdU assay. **g** The adhesion ability of the transfected U87 and U251 cells was evaluated using the cell adhesion assay. **h** The apoptosis rate of the transfected U87 and U251 cells was evaluated using flow cytometry. **i** The Twist, Slug, Vimentin, MMP-2 protein expression level of the transfected U87 and U251 cells was evaluated using western blot assay. NC, negative control. Si-LNC, si-HOXA-AS2. The cells in the blank group without any treatments. The data were presented in the form of mean ± SD, and three independent experiments were performed. *P < 0.05, **P < 0.001

**Fig. 3:**
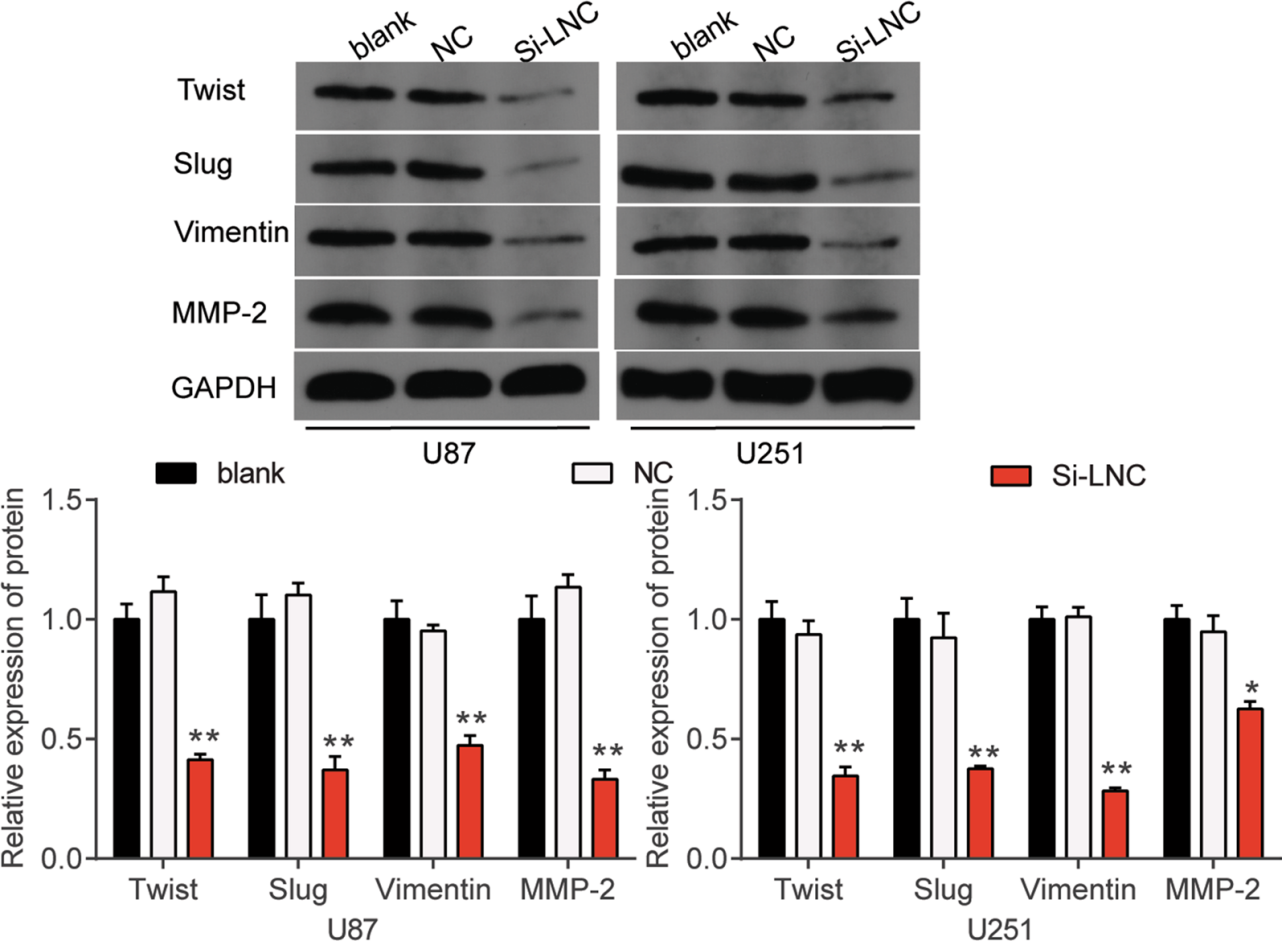
The Twist, Slug, Vimentin, MMP-2 protein expression level of U87 and U251 cells transfected with si-HOXA-AS2 was evaluated using western blot assay. NC, negative control. Si-LNC, si-HOXA-AS2. The cells in the blank group without any treatments. The data were presented in the form of mean ± SD, and three independent experiments were performed. *P < 0.05, **P < 0.001

Using qRT-PCR, we determined HOXA-AS2 levels in glioblastoma tissues and cells. The results showed that compared to adjacent healthy tissues, HOXA-AS2 expression was upregulated by twofold in glioblastoma tissues (Fig. 2a). It was also found that HOXA-AS2 expression in glioblastoma cell lines (U251, U87, A172, SHG44 and SNB19) was higher than that in the normal cell line (NHA) (Fig. 2b). Among the glioblastoma cell lines, U87 and U251 showed the highest HOXA-AS2 expression. For this reason, the two cell lines were selected in subsequent experiments. To further characterize HOXA-AS2, we explored the distribution of HOXA-AS2 in U87 and U251 cells. Our experimental results revealed that HOXA-AS2 was mainly located in the cytoplasm with low content in the nucleus (Fig. 2c). To investigate the impact of HOXA-AS2 on glioblastoma cells, we transfected si-HOXA-AS2 into U87 and U251 cells. The results of our qRT-PCR analysis showed that HOXA-AS2 silencing significantly reduced HOXA-AS2 expression by 70% compared to the blank group (Fig. 2d), meaning si-HOXA-AS2 was successfully transfected into U87 and U251 cells. CCK-8, BrdU and flow cytometry are commonly used to evaluate the proliferation and apoptosis of cancer cells [27, 28]. Our CCK-8 assay outcome indicated that compared with the blank group, silencing HOXA-AS2 inhibited cell viability after U87 and U251 cells were transfected with si-HOXA-AS2 for 48 and 72 h (Fig. 2e). The BrdU assay findings proved that si-HOXA-AS2 impaired the proliferation ability of glioblastoma cells (Fig. 2f). Adhesion is a key factor in the spread of cancer cells, and cell adhesion can be used to assess tumor development [29, 30]. Similar to cell proliferation, the cell adhesion ability was suppressed in glioblastoma cells after transfecting si-HOXA-AS2 (Fig. 2g). Besides, silencing HOXA-AS2 increased the cell apoptosis rate by almost fivefold in U87 cells and by about 7.5-fold in U251 cells (Fig. 2h). EMT-related molecules including Twist, Slug and Vimentin and MMP-2 play a crucial role in tumor metastasis and invasion [31, 32]. After examining the effect of HOXA-AS2 on the protein expression of Twist, Slug, Vimentin and MMP-2 by western blot, we found that knocking down HOXA-AS2 inhibited the protein expression of Twist, Slug, Vimentin and MMP-2 (Fig. 3).

### Si-HOXA-AS2 inhibited tumor growth in vivo

**Fig. 4:**
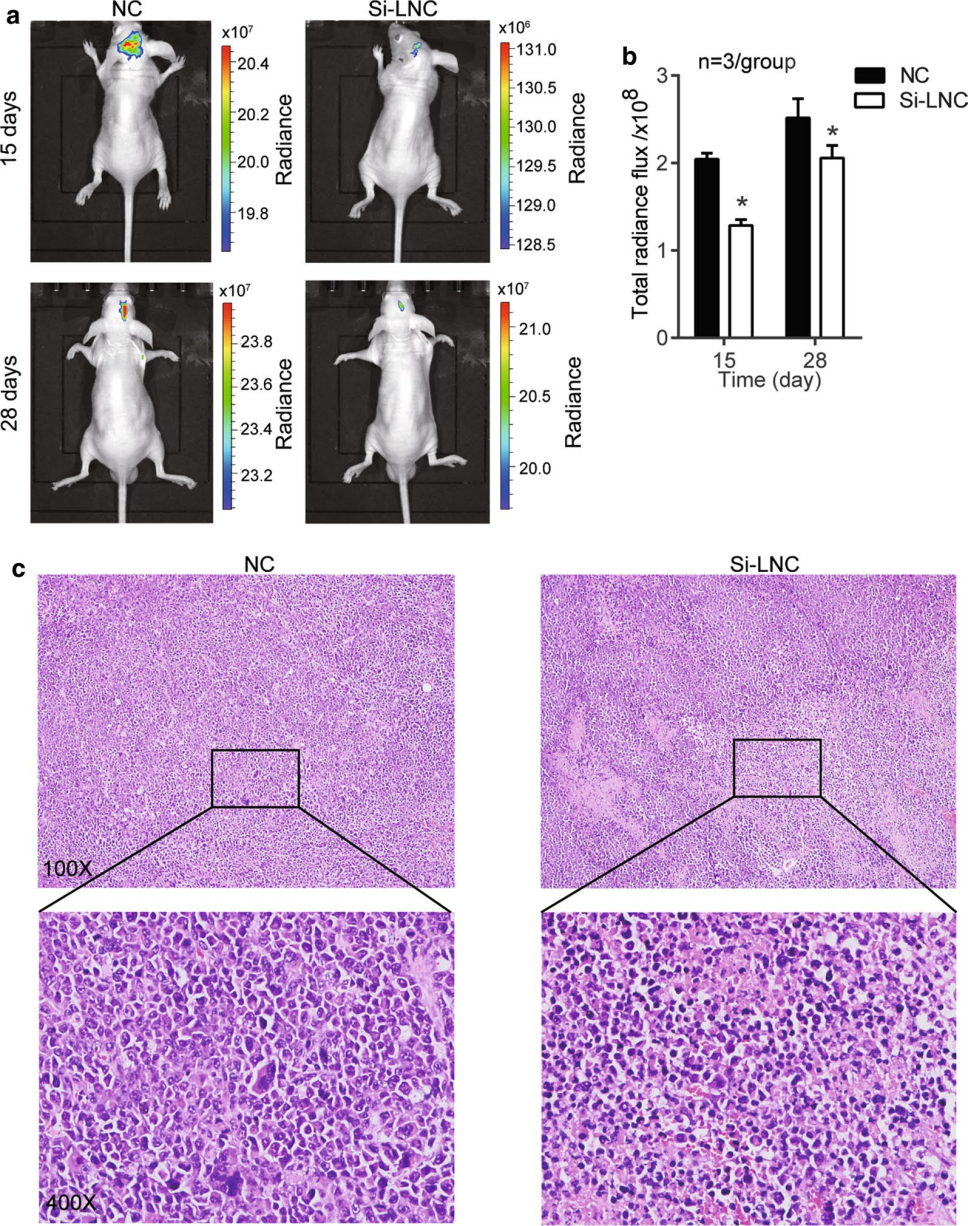
Si-HOXA-AS2 inhibited tumor growth in vivo*.* **a** The U87 cells transfected with negative control (NC) or si-HOXA-AS2 (Si-LNC) were injected into the nude mice. The representative images were obtained using an imaging system. **b** The radiance flux was monitored to represent tumor formation. **c** Representative images of HE pathological staining in xenograft tumors were obtained from the nude mice. NC, negative control. Si-LNC, si-HOXA-AS2. The data were presented in the form of mean ± SD, and three independent experiments were performed. *P < 0.05, **P < 0.001

U87 cells transfected with si-HOXA-AS2 or NC were injected into the nude mice to evaluate the effect of HOXA-AS2 on tumor growth in vivo. The results of in vivo imaging of the mice showed the total radiation flux in the HOXA-AS2 silent group was reduced compared with the negative control group (NC) (Figs. 4a, b). Hematoxylin–eosin (HE) staining showed that silencing the expression of LNC HOXA-AS2 could destroy the tumor tissue structure (Fig. 4c).

### HOXA-AS2 directly reduced miR-885-5p expression

**Fig. 5:**
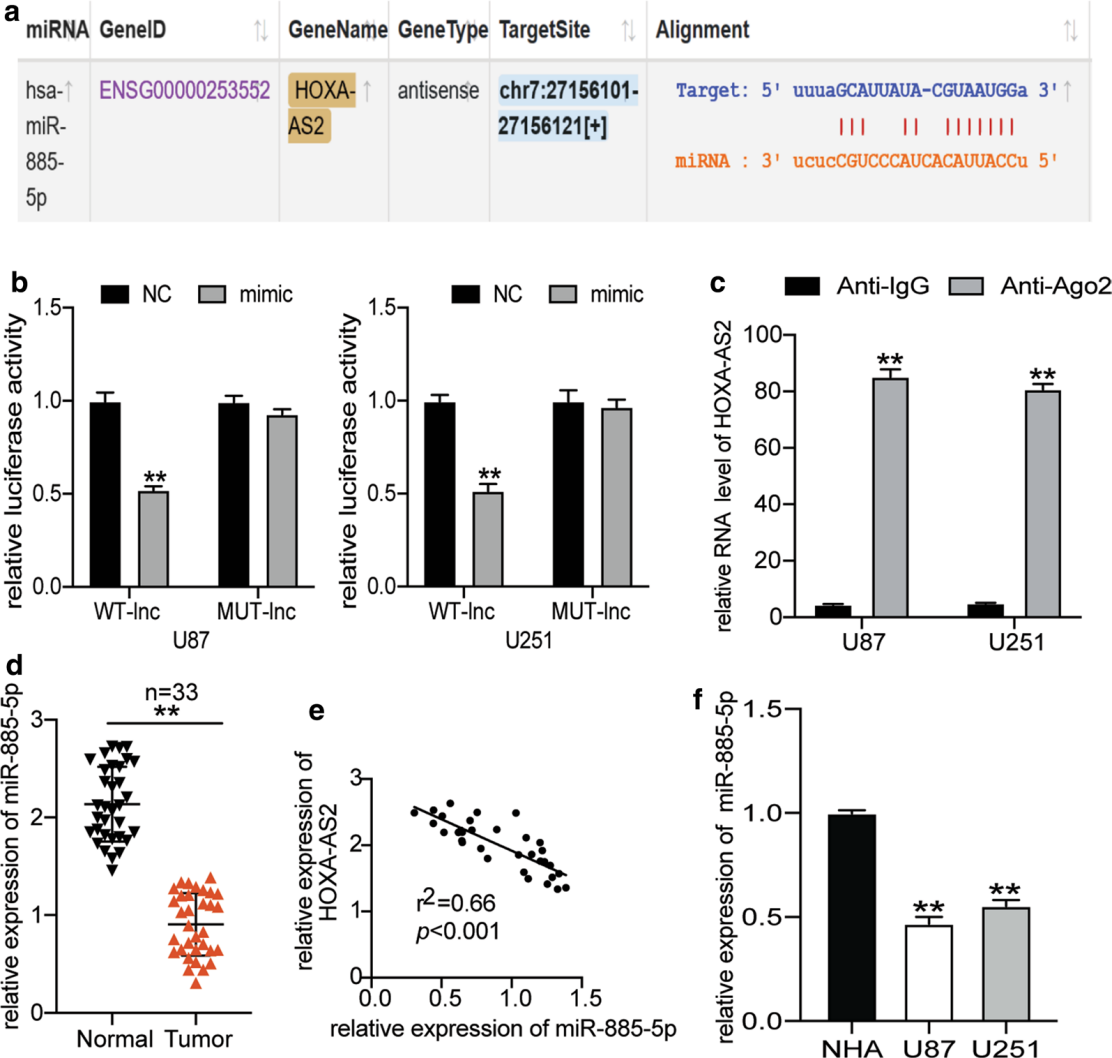
HOXA-AS2 was the target gene of miR-885-5p. **a** starBase showed the binding site between HOXA-AS2 and miR-885-5p. **b** The potential binding site between HOXA-AS2 and miR-885-5p was identified using the dual-luciferase assay. Mimic, miR-885-5p mimic. WT-lnc, wild-type HOXA-AS2. MUT-lnc, mutant HOXA-AS2. **c** The interaction between HOXA-AS2 and miR-885-5p was identified using RIP analysis. **d** MiR-885-5p expression in glioblastoma tissues and non-tumor tissues was analyzed using qRT-PCR. N = 33. **e** The correlation analysis of miR-885-5p and HOXA-AS2. **f** MiR-885-5p expression in glioblastoma cell lines (U251 and U87) and normal human astrocytes cell line (NHA) was analyzed using qRT-PCR. The data were presented in the form of mean ± SD, and three independent experiments were performed. *P < 0.05, **P < 0.001

To explore how HOXA-AS2 promoted the pathological progress of glioblastoma cells, we used starBase 3.0 (http://starbase.sysu.edu.cn/index.php) to predict the miRNAs to which HOXA-AS2 could bind. The results showed that a binding site existed between miR-885-5p and HOXA-AS2 (Fig. 5a). We then performed Dual-luciferase assay and RIP assay to verify that HOXA-AS2 could bind to miR-885-5p. The Dual-luciferase assay results showed that miR-885-5p mimic significantly reduced the fluorescence intensity of HOXA-AS2 wild-type but that it had no significant effect on the fluorescence intensity of HOXA-AS2 mutant (Fig. 5b). The RIP assay findings also showed that HOXA-AS2 and miR-885-5p interacted with each other (Fig. 5c). We later used qRT-PCR to determine the expression of miR-885-5p in glioblastoma tissues and found that miR-885-5p was not only 50% downregulation in glioblastoma tissues (Fig. 5d) but also negatively correlated with HOXA-AS2 in glioblastoma tissues (Fig. 5e). In the glioblastoma cells, miR-885-5p expression was also downregulated by approximately 50% in U87 and U251 cells (Fig. 5f).

### MiR-885-5p suppressed the malignant phenotype of glioblastoma cells

**Fig. 6:**
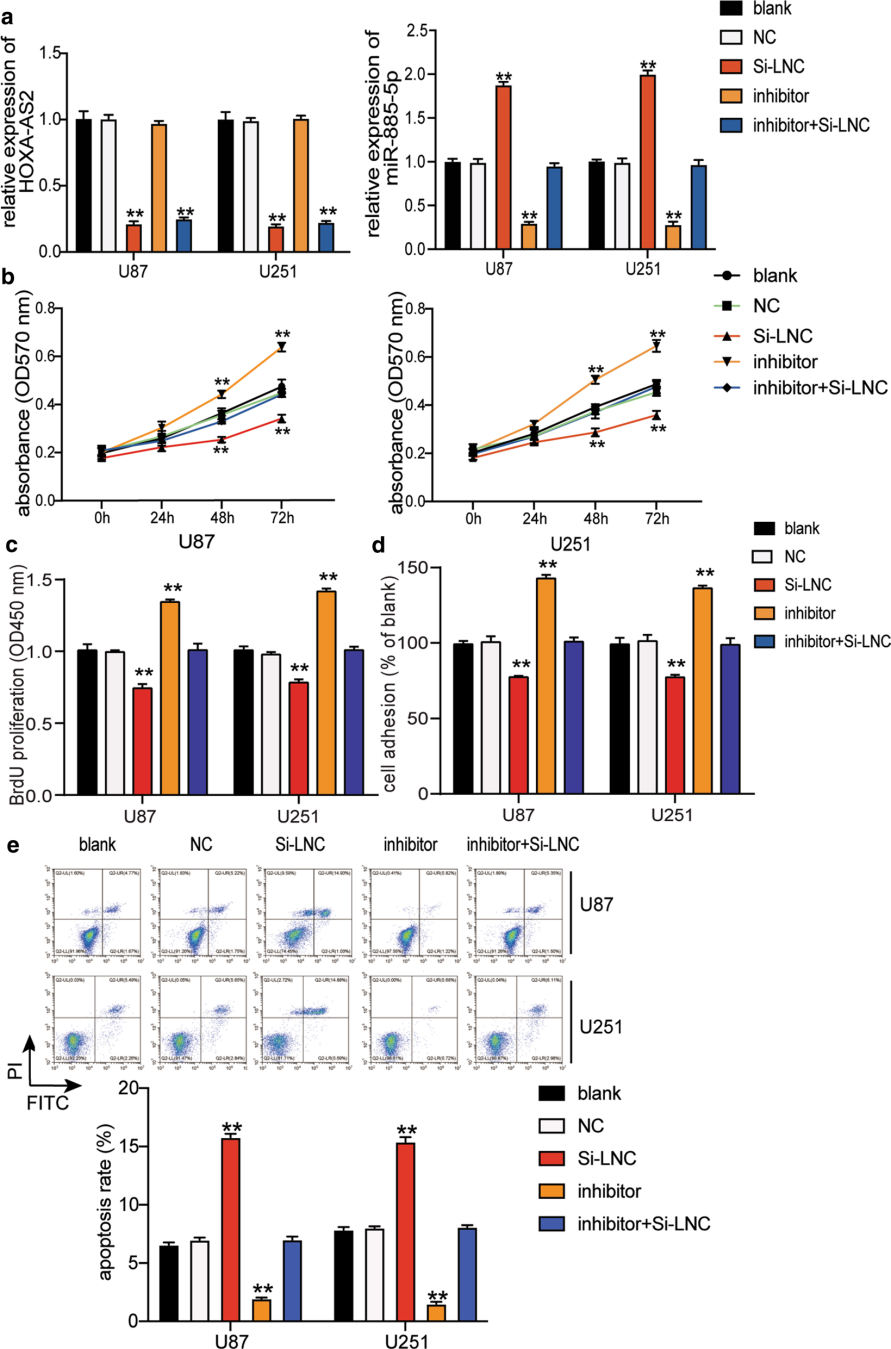
MiR-885-5p regulated by HOXA-AS2 could suppress the malignant phenotype of glioblastoma cells. **a** The transfection efficiency of si-HOXA-AS2 and miR-885-5p inhibitor was verified using qRT-PCR. **b** The viability ability of the transfected U87 and U251 cells was measured using CCK-8 assay. **c** The proliferation ability of the transfected U87 and U251 cells was measured using BrdU assay. **d** The adhesion ability of the transfected U87 and U251 cells was detected using cell adhesion assay. **e** The apoptosis ability of the transfected U87 and U251 cells was assessed using flow cytometry. The cells in the blank group without any treatments. The data were presented in the form of mean ± SD, and three independent experiments were performed. *P < 0.05, **P < 0.001

**Fig. 7:**
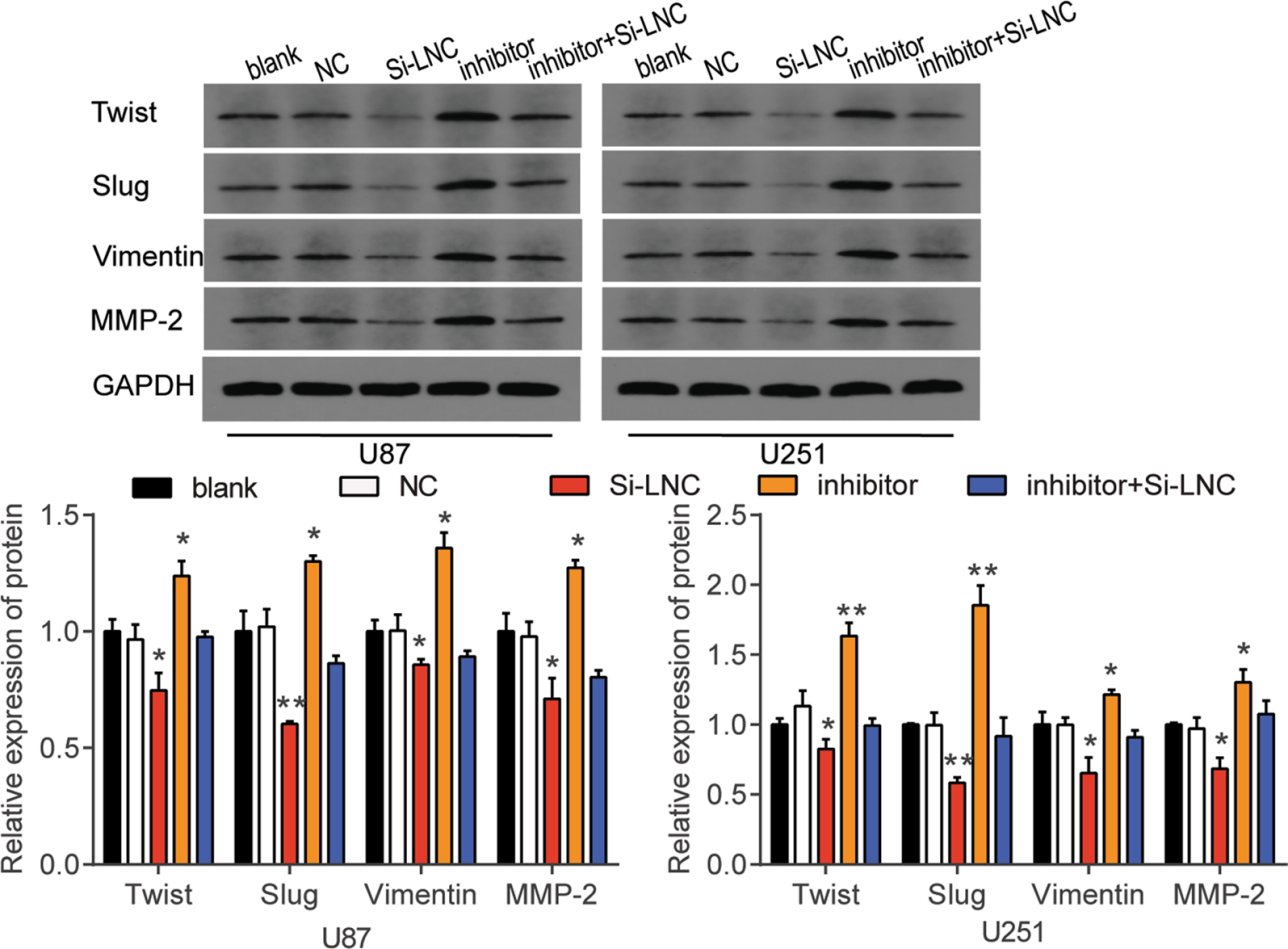
The Twist, Slug, Vimentin, MMP-2 protein expression level of U87 and U251 cells transfected with si-HOXA-AS2 or miR-885-5p inhibitor was evaluated using western blot assay. NC, negative control. Si-LNC, si-HOXA-AS2. Inhibitor, miR-885-5p inhibitor. The cells in the blank group without any treatments. The data were presented in the form of mean ± SD, and three independent experiments were performed. *P < 0.05, **P < 0.001

To investigate the effect of miR-885-5p downregulated by HOXA-AS2 on the malignant phenotype of glioblastoma cells, we transfected si-HOXA-AS2, miR-885-5p inhibitor and miR-885-5p inhibitor with si-HOXA-AS2 into U87 and U251 cells. As shown in Fig. 6a, si-HOXA-AS2 resulted in a 70% decrease in HOXA-AS2 expression and a twofold increase in miR-885-5p expression in U87 and U251 cells. As for the miR-885-5p inhibitor, we noticed a 70% decrease in miR-885-5p expression even though this decrease did not affect the HOXA-AS2 expression in glioblastoma cells. According to CCK-8 assay results, miR-885-5p downregulation promoted cell viability; however, by silencing HOXA-AS2, its effect on cell viability can be reversed (Fig. 6b). The outcome of the BrdU assay displayed that cell proliferation was elevated by almost 1.5-fold after the glioblastoma cells were transfected with the miR-885-5p inhibitor, while si-HOXA-AS2 can mitigate the influence of miR-885-5p inhibitor on cell proliferation (Fig. 6c). The cell adhesion assay results showed that miR-885-5p inhibitor increased cell adhesion by nearly 1.5 times and that si-HOXA-AS2 reversed the promotive effect of the miR-885-5p inhibitor in cell adhesion (Fig. 6d). In contrast, the downregulation of miR-885-5p decreased the cell apoptosis rate by 70% in U87 and U251 cells. Besides, silencing HOXA-AS2 attenuated the inhibitory function of the miR-885-5p inhibitor in cell apoptosis (Fig. 6e). However, the downregulation of miR-885-5p promoted the expression of Twist, Slug, Vimentin and MMP-2 proteins, while the knockout of HOXA-AS2 could eliminate the positive effect of the miR-885-5p inhibitor on these proteins expression (Fig. 7).

### MiR-885-5p combination of RBBP4 3′UTR to target RBBP4

**Fig. 8:**
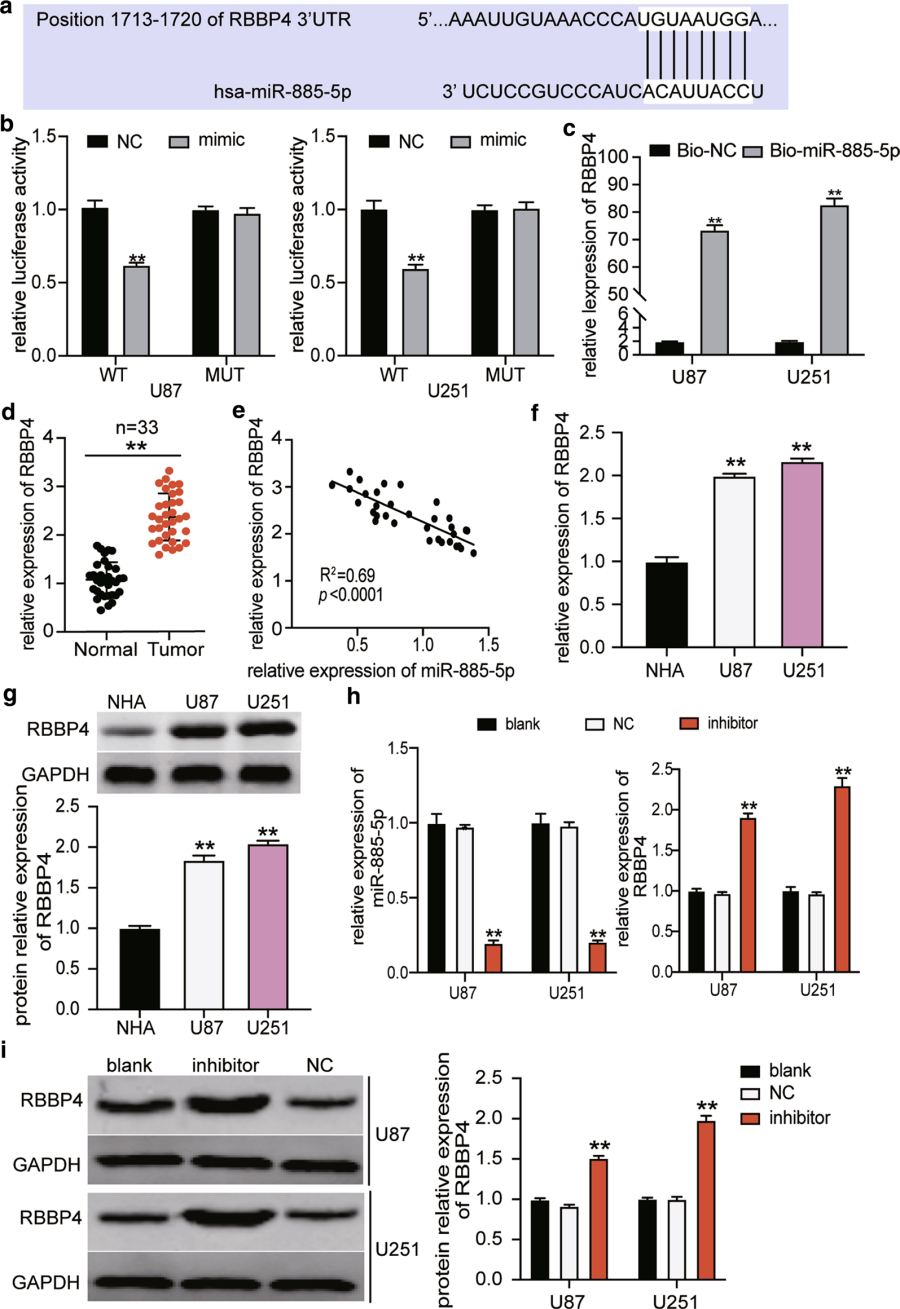
MiR-885-5p directly targeted RBBP4 by binding to its 3′UTR. **a** The potential binding site between miR-885-5p and RBBP4 was predicted using TargetScan Human 7.2. **b** The potential binding site between miR-885-5p and the 3′UTR of RBBP4 was demonstrated using the dual-luciferase assay. NC, negative control. Mimic, miR-885-5p mimic. **c** The interaction between RBBP4 and miR-885-5p was evaluated using the RNA pull-down assay. Bio-NC, biotinylated negative control. Bio-miR-885-5p, biotinylated miR-885-5p. **d** RBBP4 expression in glioblastoma tissues and normal tissues was detected by qRT-PCR. N = 33. **e** The correlation analysis of miR-885-5p and RBBP4. **f** RBBP4 expression in glioblastoma cell lines (U251 and U87) and normal human astrocytes cell line (NHA) were identified using qRT-PCR. **g** The expression of RBBP4 protein in glioblastoma cell lines (U251 and U87) and normal human astrocytes cell line (NHA) were detected with the western blot kit. **h** The expression of RBBP4 mRNA increased by miR-885-5p inhibitor in U87 and U251 cells. **i** The expression of RBBP4 protein increased by miR-885-5p inhibitor in U87 and U251 cells. NC, negative control. Inhibitor, miR-885-5p inhibitor. The cells in the blank group without any treatments. The data were presented in the form of mean ± SD, and three independent experiments were performed. *P < 0.05, **P < 0.001

TargetScan Human 7.2 was utilized to identify the miR-885-5p target genes. We found that miR-885-5p could target the position 1713–1720 of RBBP4 3′UTR (Fig. 8a). The result of the Dual-luciferase assay demonstrated that miR-885-5p decreased the fluorescence intensity of RBBP4 wild-type plasmid by 40% without affecting the RBBP4 mutant plasmid (Fig. 8b). RNA pull-down assay results also showed that an interaction existed between RBBP4 and miR-885-5p (Fig. 8c). After analyzing the expression of RBBP4 mRNA in 33 glioblastoma tissues, we noticed that RBBP4 expression was elevated by 2.5-fold in glioblastoma tissues (Figs. 8d), and RBBP4 expression was negatively related to miR-885-5p expression in glioblastoma tissues (Fig. 8e). The expression of RBBP4 mRNA and protein in glioblastoma cells was upregulated in U87 and U251 cells (Fig. 8f, g). At the same time, qRT-PCR data showed that miR-885-5p inhibitor enhanced RBBP4 expression by around twofold (Fig. 8h). Similar to the result of mRNA, the protein level of RBBP4 in the miR-885-5p inhibitor group increased by 1.5-fold in U87 cells and 1.9-fold in U251 cells (Fig. 8i).

### Inhibitory effects of miR-885-5p on glioblastoma cells via down-regulating RBBP4

**Fig. 9:**
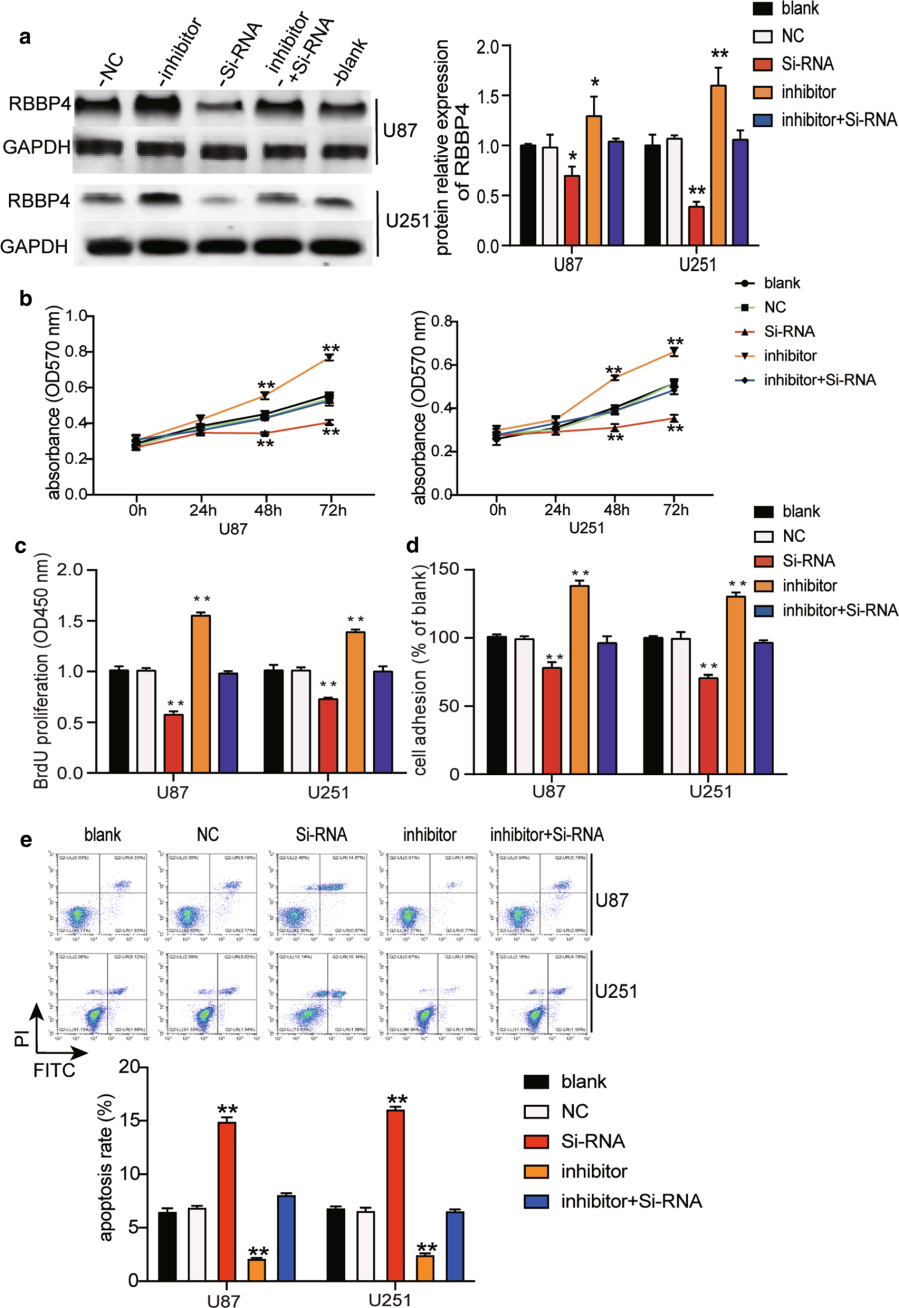
The miR-885-5p inhibited the tumorigenesis via targeting RBBP4. **a** The transfection efficiency of the miR-885-5p inhibitor and si-RBBP4 was verified with western blot assay. **b** The viability ability of the transfected U87 and U251 cells was measured with the CCK-8 assay. **c** The proliferation ability of the transfected U87 and U251 cells was measured with BrdU assay. **d** The adhesion ability of the transfected U87 and U251 cells was detected using cell adhesion assay. **e** The apoptosis ability of the transfected U87 and U251 cells was evaluated using flow cytometry. NC, negative control. Si-RNA, si-RBBP4. Inhibitor, miR-885-5p inhibitor. The data were presented in the form of mean ± SD, and three independent experiments were performed. *P < 0.05, **P < 0.001

**Fig. 10:**
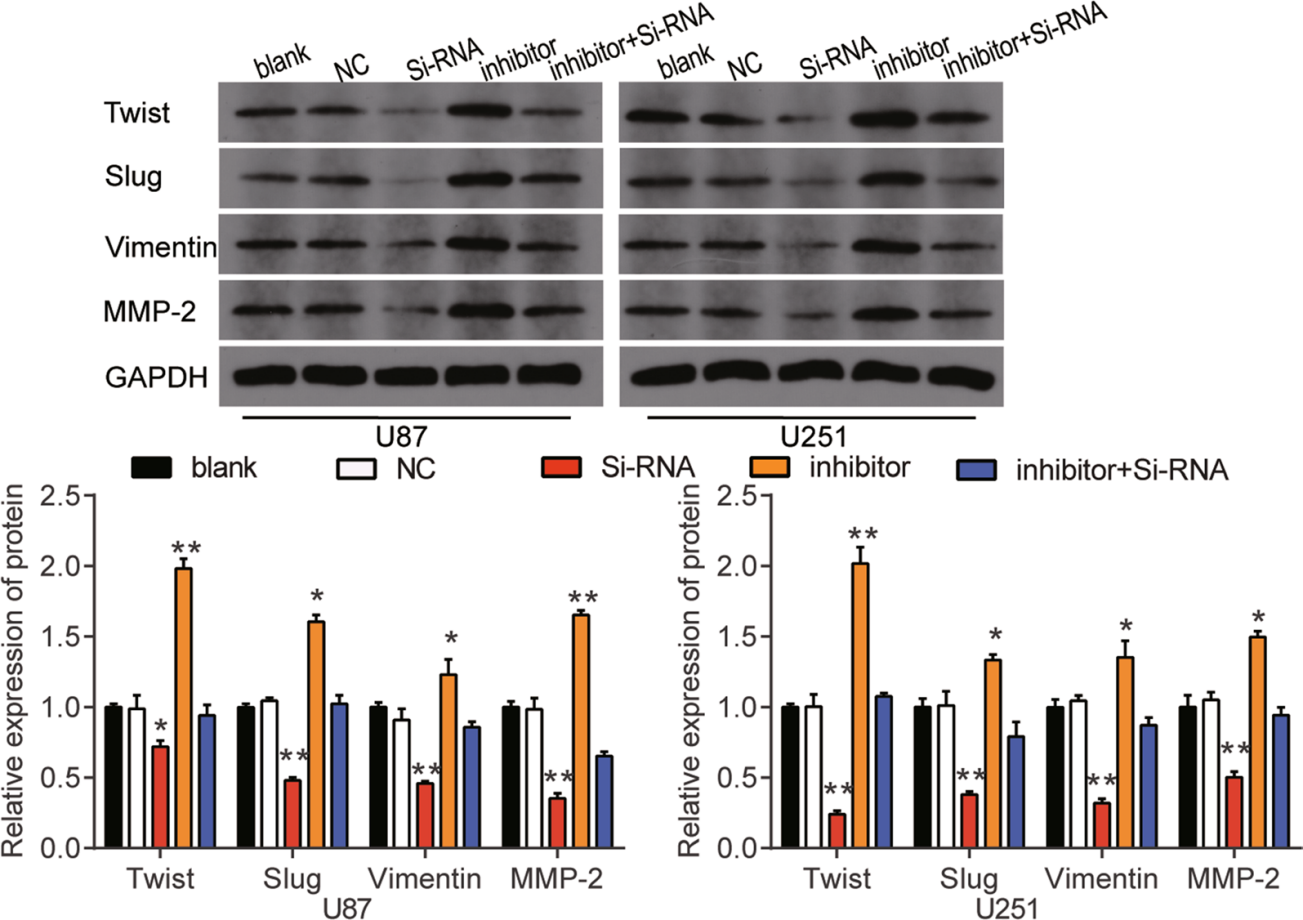
The Twist, Slug, Vimentin, MMP-2 protein expression level of U87 and U251 cells transfected with miR-885-5p inhibitor and si-RBBP4 was evaluated with western blot assay. NC, negative control. Si-RNA, si-RBBP4. Inhibitor, miR-885-5p inhibitor. The data were presented in the form of mean ± SD, and three independent experiments were performed. *P < 0.05, **P < 0.001

To investigate whether the miR-885-5p/RBBP4 axis could regulate the tumorigenesis of glioblastoma cells, we transfected si-RBBP4, miR-885-5p inhibitor or miR-885-5p inhibitor plus si-RBBP4 into U87 and U251 cells. Western blot findings showed that miR-885-5p inhibitor increased the protein level of RBBP4 by more than 1.5-fold but that si-RBBP4 reduced the protein level of RBBP4 by approximately 50% (Fig. 9a). No significant difference was observed in the protein level of RBBP4 in the co-transfection of miR-885-5p inhibitor and si-RBBP4 group compared to the blank group. The CCK-8 assay results proved that si-RBBP4 inhibited the cell viability of the glioblastoma cells after a transfection period of 48 and 72 h. Also, si-RBBP4 can eliminate the cell viability abilities of the miR-885-5p inhibitor (Fig. 9b). The result of the BrdU assay was consistent with that of the CCK-8 assay. In other words, si-RBBP4 played an inhibitory role in cell proliferation, and it eliminated the promotive effect of the miR-885-5p inhibitor on cell proliferation (Fig. 9b). The cell adhesion assay outcome revealed that si-RBBP4 decreased cell adhesion by around 30% compared to the blank group. Moreover, si-RBBP4 could counteract the cell adhesion role of the miR-885-5p inhibitor in glioblastoma cells (Fig. 9d). Apart from that, the results of the flow cytometry analysis showed that the cell apoptosis rate of both U87 and U251 cells increased by 2.5-fold, and the inhibitory cell apoptosis rate caused by miR-885-5p inhibitor could be overturned by si-RBBP4 (Fig. 9e). Western blot assay results showed that the interference of RBBP4 decreased the protein expression of Twist, Slug, Vimentin and MMP-2 in U87 and U251 cells, and reverse the positive effect of the miR-885-5p inhibitor on these proteins expression (Fig. 10).

## Discussion

CCK-8 assay, BrdU assay and flow cytometry are commonly used to study the proliferation and apoptosis of cancer cells [27, 28]. Cell adhesion assay is also performed to assess tumor development [29, 30]. EMT-related molecules (Twist, Slug, Vimentin and MMP-2) even play an important role in tumor metastasis and invasion [31, 32]. To explore the progression of glioblastoma cells, we detected cell proliferation, apoptosis, and adhesion, as well as the EMT related proteins levels. In this study, HOXA-AS2 was found to be overexpressed in glioblastoma tissues and cells, to inhibit cell apoptosis, and to enhance cell proliferation, migration, invasion and the protein expression of Twist, Slug, Vimentin and MMP-2. We also noticed that miR-885-5p, which was directly inhibited by HOXA-AS2, was downregulated during the pathological process of glioblastoma. It also repressed the growth of glioblastoma cells. RBBP4, a measurable downstream target of miR-885-5p, was found to be upregulated in glioblastoma cells, thereby enhancing the malignant phenotypes of glioblastoma cells. Overall, HOXA-AS2 promoted glioblastoma through the HOXA-AS2/miR-885-5p/RBBP4 axis.

Furthermore, HOXA-AS2 was found to be upregulated and to exert the pro-oncogenic function in cancer processes. For instance, in one study, HOXA-AS2 stimulated the proliferation, migration and invasion of osteosarcoma cells [11]. Several studies described HOXA-AS2 as an oncogene that promoted the malignant proliferation of non-small cell lung cancer cells [33–35]. Similarly, HOXA-AS2 was demonstrated in another research to advance bladder cancer cells by directly regulating the expression of miR-125b [36]. Furthermore, HOXA-AS2 was identified to promote breast cancer cells via the HOXA-AS2/miR-106a/SCN3 axis [37]. In glioma cells, HOXA-AS2 recruits EZH2 to upregulate RND3 and promote the proliferation of glioma cells, thereby accelerating tumor growth [13]. Our study showed that HOXA-AS2 upregulation occurred in glioblastoma tissues, thereby increasing the aggressiveness of glioblastoma cells. In contrast to the results of previous studies on HOXA-AS2, our study showed that HOXA-AS2 increased RBBP4 by targeting miR-885-5p to enhance the viability of malignant cells.

In addition, miR-885-5p has been proved to exert different effects in different cancers due to its abnormal expression in multiple cancer types. For instance, miR-885-5p inhibited the proliferation, invasion and angiogenesis abilities of hepatocellular carcinoma cells by targeting AEG1 [38]. In gastric cancer, the miR-885-5p expression accelerated the metastasis of gastric cancer cells, and the cell functional experiments proved that the downregulation of miR-885-5p inhibited the proliferation, colony formation and invasion of gastric cancer cells [39]. In their research on glioblastoma, Yan et al*.* used a miRNA microarray in 60 glioblastoma multiforme samples to show that miR-885-5p has a high correlation with MMP-9 expression [20]. What’s more, in a study conducted by Yan et al*.*, miR-885-5p overexpression was discovered to reduce the MMP-9 level, thereby suppressing the invasion abilities of glioma cells. Another study found that miR-885-5p overexpression restrained the development of gliomagenesis [19]. In our study, miR-885-5p was predicted to be the downstream miRNA of HOXA-AS2. After carrying out CCK-8 assay, BrdU assay, cell adhesion assay, flow cytometry, and western blot assay, we noticed that miR-885-5p inhibited the malignant phenotypes of glioblastoma cells. Our result was consistent with the previous studies on miR-885-5p in glioma cells.

We identified and analyzed the predicted target genes of miR-885-5p from the miRDB.org database and the DEGs from GEPIA. The intersected genes were uploaded to STRING for the potential protein–protein interaction analysis. We found that RBBP4 showed a very high confidence level. In the literature review, it was reported that RBBP4, a subunit of the NURF complex, facilitated hepatocellular carcinoma by interacting with other components to silence tumor suppressor genes [40]. In another study, RBBP4 silencing impaired the proliferation of gastric cancer cells but stimulated apoptosis [23]. These findings provided conclusion that RBBP4 might be an oncogene for cancer progression. After performing bioinformatics analysis, RBBP4 was identified to be the key gene in glioblastoma. The results of the Dual-luciferase assay and RNA pull-down assay even confirmed that RBBP4 to be the target gene of miR-885-5p and that it could suppress RBBP4 expression. Besides, the RBBP4 served as an oncogene to facilitate malignant phenotypes of glioblastoma cells, and it could overturn the inhibitory influence of miR-885-5p on glioblastoma cells.

In short, our study proved that the HOXA-AS2/miR-885-5p/RBBP4 axis influenced the growth of glioblastoma cells. However, our studies have some limitations. This research did not study the downstream signaling pathway of RBBP4. This limitation makes it difficult to understand the impact of the HOXA-AS2/miR-885-5p/RBBP4 axis on glioblastoma progression. Another limitation is the small sample size used in this study. We believe that a large sample size would improve the accuracy of the results.

## Conclusion

Our research revealed the influence of the HOXA-AS2/miR-885-5p/RBBP4 axis on the progression of glioblastoma carcinogenesis. More specifically, we found that HOXA-AS2 contributed to the viability, proliferation and adhesion of glioblastoma cells, but it inhibited cell apoptosis by stimulating miR-885-5p to release RBBP4. Our findings may help provide the theoretical basis and understanding of glioblastoma treatments.

## Data Availability

The datasets used during the current study are available from the corresponding author on reasonable request.

## Abbreviations

miRNAs: MicroRNAs
PBS: Phosphate buffered solution
EdU: 5-Ethynyl-2′-deoxyuridine
HOXA-AS2: HOXA cluster antisense RNA 2
qRT-PCR: Quantitative real-time PCR
